# Risks of urgent cesarean delivery preceding the planned schedule: A retrospective cohort study

**DOI:** 10.1371/journal.pone.0289655

**Published:** 2023-08-07

**Authors:** Shir Danieli-Gruber, Yael Shalev-Rosenthal, Ran Matot, Noa Brzezinski-Sinai, Gil Zeevi, Anat Pardo, Sharon Orbach, Eran Hadar

**Affiliations:** 1 Helen Schneider Hospital for Women, Rabin Medical Center – Beilinson Hospital, Petach Tikva, Israel; 2 Sackler Faculty of Medicine, Tel Aviv University, Tel Aviv, Israel; Werabe University, ETHIOPIA

## Abstract

**Purpose:**

The aim of the study was to ascertain risk factors and outcomes of elective cesarean deliveries performed urgently prior to their scheduled date.

**Methods:**

Women carrying a viable singleton fetus who were scheduled for elective cesarean delivery at a tertiary medical center between 2012–2020 were identified by retrospective database. Differences in maternal and neonatal parameters between those who ultimately required urgent cesarean delivery and those who underwent the procedure as scheduled were analyzed.

**Results:**

Of 4403 women who met the inclusion criteria, 559 underwent urgent cesarean delivery before the scheduled date. On multivariate analysis, factors significantly associated with a risk of transformation to an urgent cesarean delivery were chronic hypertension (aOR 1.92, 95% CI 1.30–2.83 P = 0.001), antenatal corticosteroids administration (aOR 3.26, 95% CI 2.38–4.47, P <0.001), and contraindication for vaginal delivery as the reason for elective cesarean delivery (aOR 1.67, 95% CI 1.32–2.12, P <0.001). Neonates born via urgent cesareans had an increased risk of 1-minute Apgar <7 (6% vs. 1.7%, P <0.001), intensive care unit admission (6.6% vs. 2.5%, P <0.001); their mothers were at risk of postpartum hemorrhage (5.9% vs. 3%, P = 0.001).

**Conclusions:**

The present study sheds light on the risk factors and maternal and fetal morbidities associated with elective cesarean deliveries that become urgent before the originally scheduled date. Physicians should take this information into account when planning an optimal date for elective cesarean delivery.

## Introduction

Cesarean delivery (CD) is one of the most prevalent surgical procedures performed worldwide. In recent years, the rate of CD has almost doubled [1, 2] with studies in the United States reporting an increase from 16 million births in 2000 to 29.7 million in 2015 [3].

Urgent CDs are performed in the presence of various concerning indications such as non-reassuring fetal heart rate, labor dystocia, or cephalopelvic disproportion, where continuation of pregnancy may jeopardize maternal and neonatal outcomes [4–6]. Planned elective CD is recommended when it is likely to provide a better maternal or fetal outcome than vaginal delivery [4]. Accepted medical and obstetrical indications for elective CD include past cesarean delivery, fetal malpresentation, and multiple gestation, although indications may slightly differ according to local national guidelines.

When scheduling an elective CD, physicians need to carefully weigh the maternal and neonatal risks and benefits. On the one hand, CD is best performed at late term when neonatal complications are low [7–10]. Studies have shown that the risk of respiratory distress syndrome and transient tachypnea of the newborn decreases as gestational age of planned CD increases from 37 to 40 weeks [11–14]. On the other hand, elective CD should be done as early as possible to avoid the onset of spontaneous labor which would necessitate unscheduled urgent CD. Maternal morbidity is higher in urgent than in elective CD due to pelvic organ injury, hemorrhage, need for blood transfusions, wound site complications, and longer hospitalization [15, 16]. The National Institute of Health and Care Excellence and the American College of Obstetricians and Gynecologists recommend scheduling elective CD at 39–40 gestational weeks unless there is an obstetric or medical indication for earlier delivery [17, 18].

Numerous studies have examined the indications and outcomes of planned and elective CDs. However, factors that play a role when a CD that was preplanned becomes urgent have hardly been investigated. The aim of this study was to ascertain the risks, indications, and maternal-neonatal outcomes of elective CDs that precede their original scheduled date in order to identify the patient group that may require more careful planning.

## Materials and methods

### Design and patients

A retrospective cohort design was used. The database of a university-affiliated tertiary medical center was searched for all women carrying a viable singleton fetus who were scheduled for elective CD between July 2012 and December 2020. Women who had a vaginal delivery, late abortion, or preterm birth (before 24 gestational weeks) were excluded as were women with a fetus with congenital anomalies or multiple gestation or who underwent elective CD prior to 37 or later than 40 gestational weeks. The women eligible for the study were divided into two groups: those who underwent urgent CD before the preplanned CD date and those who underwent planned elective CD as scheduled (controls).

### Data collection

Maternal, obstetric, surgical, and neonatal parameters were derived from the department’s comprehensive electronic medical records as follows: maternal age, height, and weight; gravidity, parity, use of assisted reproductive techniques (ART), and previous CD; pregnancy complications such as hypertensive disorders in pregnancy, diabetes mellitus in pregnancy (type 1, type 2 or gestational), antenatal corticosteroids administration, adherent placenta, and placenta previa; intrapartum characteristics including indications for planned and urgent CD, type of anesthesia, duration of surgery, and number of days between scheduled and actual delivery dates; intraoperative and postoperative complications such as intra-abdominal adhesions, bladder lacerations, uterine scar dehiscence or rupture, cesarean hysterectomy, postpartum hemorrhage, surgical site infection, paralytic ileus and number of hospitalization days. For women who underwent urgent CD ahead of the planned date, the indications leading to the pre-scheduled CD were categorized as term or preterm premature rupture of membranes (PROM or PPROM), preterm contractions and cervical dynamics, non-reassuring fetal heart rate (NRFHR), chorioamnionitis, and placental abruption. Neonatal outcome parameters included gestational age at birth, birthweight, 1- and 5-min Apgar scores, umbilical cord blood pH, and neonatal intensive care unit (NICU) admission.

### Definitions

Gestational age was determined by the last menstrual period and affirmed by crown-rump length measurement on the first-trimester ultrasound scan. Small for gestational age was defined as birthweight below the 10th percentile according to nationally accepted birthweight reference curves [19].

Elective planned CD was defined as CD performed prior to the expected due date for any of the following indications: 1) past uterine surgery, 2) relative or absolute contraindications for vaginal birth, including malpresentation, suspected macrosomia, fetal growth restriction, labial herpes virus infection, maternal request; (3) others, such as maternal orthopedic/ophthalmic medical conditions.

At our institution, elective CD is scheduled in a pre-op ambulatory meeting held 8–10 weeks before the accepted due date, after review of the full patient history and results of physical examination and antenatal follow up. First or second CDs are usually scheduled for 39 gestational weeks, according to accepted guidelines [17, 18]; third or more CDs are scheduled for 38–39 gestational weeks. In cases of placenta previa, previous uterine surgeries, or prior intrauterine fetal death, elective CD is routinely scheduled for 37–38 gestational weeks.

### Outcome measures

The main outcome of the study was to identify factors associated with early performance of elective CD, before the scheduled date. Secondary outcome measures were maternal and neonatal outcomes of urgent CD in this setting.

### Ethical approval

Research involving human subjects complied with all relevant national regulations, institutional policies and is in accordance with the tenets of the Helsinki Declaration (as revised in 2013), and has been approved by the authors’ Institutional Review Board (Rabin Medical Center Helsinki Review Board, approval no. 0087-21-RMC), with waiver of informed consent due to the retrospective, observational design of the study.

### Statistical analysis

Statistical analysis was performed with SAS version 9.4 (Cary, NC, USA). Continuous variables were compared between groups using the general linear model (GLM); chi-square and Fisher’s exact tests were used for categorical variables, as appropriate. Differences were considered significant when p was less than 0.05. To identify factors affecting the risk of pre-scheduled performance of elective CD, multivariate analysis was used, adjusted for confounders.

## Results

**Fig 1:**
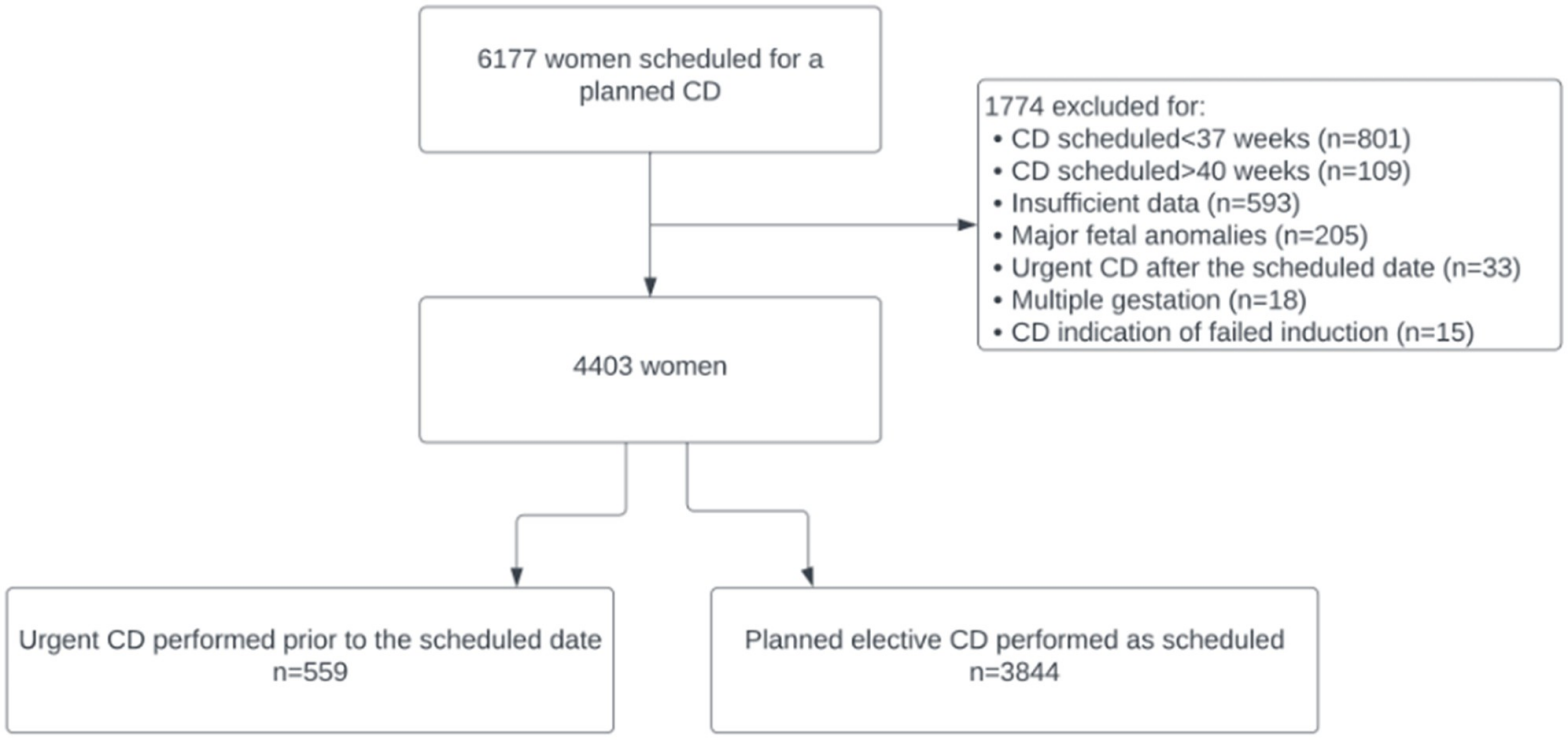
Flowchart of patients’ cohorts and exclusion criteria.

Of the 6177 women who were scheduled for a planned CD during the study period, 1774 were excluded for the following reasons (Fig 1): CD scheduled for earlier than 37 gestational weeks (n = 801) or after 40 gestational weeks (n = 109), insufficient data (n = 593), major fetal anomalies (n = 205), urgent CD performed after the scheduled date (n = 33), multiple gestation with or without selective fetal reduction (n = 18), and CD due to failed induction (n = 15). The remaining 4403 women formed the final cohort, of whom 559 (13%) had an urgent CD preceding the planned schedule and 3844 (87%) underwent the procedure as scheduled (control group). The final cohort included women carrying a viable singleton fetus who were scheduled for an elective CD between 37 to 40 weeks.

The baseline characteristics of the two groups are presented in Table 1. Compared to controls, women who required an urgent CD were more often nulliparous (22.4% vs. 16.9%, P = 0.002) and had higher rates of chronic hypertension (7.5% vs. 3.5%, P<0.001), pregestational diabetes (5.9% vs 3.3%, P = 0.05), hypertensive disorders of pregnancy (5.7% vs. 1.9%, P <0.001), and ART (12.6% vs. 9.4%, P = 0.046), and a lower rate of prior CD (60.8% vs. 66.3%, P = 0.012).

**Table 1:** Baseline characteristics of women who underwent urgent or elective cesarean delivery.

Characteristics	Urgent cesarean delivery	Elective cesarean delivery	P value^a^
	n = 559	n = 3 844	
**Demographics and clinical variables**			
Maternal age (years)	34 (30–38)	34 (31–38)	0.68
Body mass index (kg/m2)			
Underweight <18.5	25 (4.47)	123 (3.2)	0.83
Normal weight 18.5–25	199 (35.6)	1168 (30.4)	
Overweight 25–30	96 (17.2)	562 (14.6)	
Obese >30	68 (12.2)	425 (11.1)	
Gestational weight gain (kg)	10.1 (7–14.5)	10.7 (6–13)	0.08
Gravidity	3.1 (2–4)	3.2 (2–3)	0.01
Parity	1.4 (1–2)	1.5 (1–2)	0.002
Nulliparity	125 (22.4)	648 (16.9)	0.002
**Medical history**			
Number of prior CDs	0.94 (0–2)	1.07 (0–2)	0.002
Any prior CD	340 (60.8)	2 548 (66.3)	0.012
Chronic hypertension	42 (7.5)	136 (3.5)	<0.001
Type 1 or type 2 diabetes	33 (5.9)	128 (3.3)	0.005
Inherited thrombophilia	10 (1.8)	63 (1.6)	0.72
Epilepsy	5 (0.9)	20 (0.5)	0.23
Hypothyroidism	34 (6.1)	222 (5.7)	0.77
Hyperthyroidism	4 (0.72)	29 (0.75)	>0.99
Anti-phospholipid syndrome	13 (2.3)	48 (1.2)	0.05
Systemic lupus erythematosus	3 (0.5)	12 (0.3)	0.42
Asthma	18 (3.2)	140 (3.6)	0.71
**Mode of conception**			
Spontaneous	488 (87.2)	3 484 (90.6)	0.046
COH	9 (1.6)	45 (1.2)	
IVF	62 (11.1)	315 (8.2)	
**Diseases of pregnancy and complications**			
Gestational diabetes mellitus	76 (13.6)	611 (15.8)	0.17
Hypertensive disorders of pregnancy^b^	32 (5.7)	73 (1.9)	<0.001

Values are presented as median (interquartile range) for continuous variables and n (%) for categorical variables.

^a^ Normally distributed variables were compared using t-test, and non-normally distributed variables were compared using Wilcoxon test.

^b^ Defined as any of the following: gestational hypertension, mild and severe preeclampsia, eclampsia, hemolysis, elevated liver enzymes and low platelets (HELLP) syndrome.

CD, cesarean delivery; COH, controlled ovarian hyperstimulation; IVF, in vitro fertilization

The birth characteristics of the two groups are presented in Table 2. The median gestational age (and range) at actual delivery was earlier in the urgent CD group compared to elective CD (37+4 (36+6–38+1) vs. 38+2 (37+6–38+5), <0.001). The median interval between the urgent CD and the scheduled CD date was 5 days (0–51). The most common indications for urgent CD among the women scheduled for elective CD were contractions (42.6%), PROM and PPROM (30.0%), and NRFHR (7.7%). The indications for elective CD differed between the groups. Contraindication for vaginal delivery was a more prevalent indication for elective CD in the urgent CD group than the control group (40.1% vs. 24.5%, P<0.001), whereas prior CD was a more prevalent indication in the control group (66.0% vs. 57.9%, P = 0.002). The urgent-CD group was more likely to have received antenatal corticosteroids treatment (11.8% vs. 3.9%, P<0.001) and had a higher median gestational age at antenatal corticosteroids administration (33+0[24+6–36+4] vs. 31+4[23+6–36+6], P = 0.007). Rates of placental abruption, contractions, NRFHR, PPROM, PROM, meconium-stained amniotic fluid, placenta previa, polyhydramnios, oligohydramnios (P<0.05 for all), and uterine rupture (P<0.01) were all higher in the urgent-CD group (Table 2). Median operative time was slightly longer in the urgent-CD group (34[16–180] vs. 31[16–260] minutes, P<0.001).

**Table 2:** Birth characteristics of women who underwent urgent or elective cesarean delivery.

Characteristics	Urgent cesarean delivery	Elective cesarean delivery	P value ^a^
	n = 559	n = 3 844	
Planned gestational age at delivery (weeks)	38.43 (38–38.86)	38.29 (37.86–38.7)	<0.001
Actual gestational age at delivery (weeks)	37.57 (36.86–38.14)	38.29 (37.86–38.71)	<0.001
Planned to actual delta (days)	5 (2–11)	NA	-
**Indications for elective cesarean delivery**			
Prior uterine surgeries	324 (57.90)	2 537 (66)	<0.001
Contraindication for vaginal delivery	224 (40.1)	942 (24.5)	<0.001
Other	11 (1.97)	365 (9.5)	<0.001
**Indications for urgent cesarean delivery**			
Contractions	238 (42.6)	NA	-
PROM/PPROM	168 (30)		
NRFHR	43 (7.7)		
Other	110 (19.5)		
**Prenatal management and complications**			
antenatal corticosteroids administration	66 (11.8)	149 (3.9)	<0.001
Gestational age at antenatal corticosteroids administration	32.79 (31.79–33.29)	33.29 (31.43–33.71)	0.007
antenatal corticosteroids to delivery interval, days	18.5 (3–39)	42 (30–59)	<0.001
Adherent placenta	7 (1.25)	41 (1.1)	>0.99
Chorioamnionitis	2 (0.4)	2 (0.05)	0.081
NRFHR	43 (7.7)	8 (0.2)	<0.001
Meconium-stained amniotic fluid	34 (6.1)	49 (1.3)	<0.001
Polyhydramnios	30 (5.4)	103 (2.7)	0.001
Oligohydramnios	21 (3.8)	57 (1.5)	<0.001
PROM, preterm	36 (6.4)	37 (1.0)	<0.001
PROM, term	105 (18.8)	91 (2.4)	<0.001
Contractions, term	144 (25.8)	23 (0.6)	<0.001
Contractions, preterm	7 (1.25)	0 (0)	<0.001
Placental abruption	9 (1.6)	21 (0.55)	0.01
Placenta previa	31 (5.5)	137 (3.6)	0.03
**Surgical parameters**			
Surgery time (minutes)	34 (28–44)	31 (25–41)	<0.001
Anesthesia			
General	65 (11.6)	165 (4.3)	<0.001
Spinal	494 (88.4)	3679 (95.7)	>0.99
**Intraoperative complications**			
Uterine scar dehiscence	14 (2.5)	87 (2.3)	0.65
Uterine scar rupture	9 (1.6)	5 (0.1)	<0.001
Uterine incision extension	1 (0.2)	8 (0.2)	>0.99
Urinary bladder injury	3 (0.5)	6 (0.2)	0.09
Intra-abdominal adhesions	17 (3.0)	86 (2.2)	0.23
Hysterectomy	2 (0.04)	2 (0.05)	0.08

Values are presented as median (interquartile range) for continuous variables and as n (%) for categorical variables.

^a^ Normally distributed variables were compared using t-test, and non-normally distributed variables were compared using Wilcoxon test.

NRFHR, non-reassuring fetal heart rate; PROM, premature rupture of membranes

Postpartum maternal and neonatal outcomes are shown in Table 3. Compared to controls, the urgent-CD group had higher rates of postpartum hemorrhage (5.9% vs 3.0%, P = 0.001), blood transfusions (2.5% vs. 1.1% P = 0.012), magnesium administration (3.2% vs 0.6%, P<0.001), NICU admission (6.6% vs. 2.5%, P<0.001), and 1-minute Apgar <7 (6% vs. 1.7%, P<0.001).

**Table 3:** Neonatal and postpartum maternal outcome of women who underwent urgent or elective cesarean delivery.

Characteristics	Urgent cesarean delivery	Elective cesarean delivery	P value ^a^
	n = 559	n = 3 844	
**Neonatal**			
Birth weight (grams)	2 951 (2 627–3 286)	3 201 (2 910–3 460)	<0.001
Neonatal gender, male	295 (52.8)	1 961 (51.0)	0.44
Small for gestational age	27 (4.8)	159 (4.1)	0.43
1-minute Apgar score <7	34 (6.1)	67 (1.7)	<0.001
5-minute Apgar score <7	6 (1.07)	16 (0.4)	0.051
Umbilical arterial pH <7.2	17 (3.0)	80 (2.1)	0.16
NICU admission	37 (6.6)	96 (2.5)	<0.001
**Maternal**			
Postpartum hemorrhage	33 (5.9)	116 (3.0)	0.001
Blood transfusion	14 (2.5)	41 (1.1)	0.012
Birth to discharge (days)	4 (4–4)	3.8 (3–4)	<0.001
Maximal WBC	9.7 (8.5–14)	9.5 (8.5–13)	<0.001
Seroma	0 (0)	1 (0.0)	>0.99
Antibiotic treatment	7 (1.25)	15 (0.4)	0.016
Magnesium PE treatment	18 (3.22)	22 (0.6)	<0.001
SSI	0 (0)	8 (0.2)	0.6
Ileus	0	3 (0.1)	>0.99

Values are presented as median (interquartile range) for continuous variables and as n (%) for categorical variables.

^a^ Normally distributed variables were compared using t-test, and non-normally distributed variables were compared using Wilcoxon test.

NICU, neonatal intensive care unit; WBC, white blood cell count; PE- Preeclampsia; SSI, surgical site infection;

On multivariate analysis controlling for potential confounders (Table 4) of maternal age, nulliparity, chronic hypertension, gestational and pregestational diabetes, ART, antenatal corticosteroids administration, and elective surgery indications, chronic hypertension had an adjusted odds ratio (aOR) of 1.92 (95% CI 1.3–2.8, P = 0.001), antenatal corticosteroids treatment had an aOR of 3.26 (CI 2.3–4.4, P<0.001) and contraindication for vaginal delivery had an aOR of 1.67 (CI 1.32–2.12, P <0.001) for undergoing urgent CD. Maternal age, ART, gestational and pregestational diabetes in pregnancy, and nulliparity were no longer significant.

**Table 4:** Multivariate analysis: Factors associated with elective cesarean deliveries performed before their planned date.

Effect	Odds ratio	95% CI limits	
Maternal age at birth	0.997	0.979	1.015
Nulliparity	0.926	0.693	1.237
Chronic hypertension	1.919	1.300	2.833
Gestational diabetes, type 1 and type 2	0.996	0.788	1.258
Planned indication- contraindication for vaginal delivery vs. previous CD	1.671	1.319	2.116
Planned indication other vs. previous CD	0.208	0.111	0.391
antenatal corticosteroids administration	3.260	2.377	4.471
Spontaneous conception	0.827	0.605	1.128

CD, cesarean delivery

Stratifying the gestational age at which CD was planned by indication revealed that women with previous uterine surgery were scheduled for elective CD at an earlier median gestational age than women with a contraindication for vaginal delivery (38+1±0.64 vs 38+4±0.66 weeks, respectively, P<0.001). Women with two or more past CDs were scheduled for CD at an even earlier gestational age (38+0±0.59 weeks).

## Discussion

This study sought to characterize the population of women who are scheduled for elective CD but undergo urgent CD before the planned date. Our key findings were as follows: (1) Chronic hypertension, antenatal corticosteroids administration, and a contraindication for vaginal delivery were risk factors for urgent CD. (2) Rates of postpartum hemorrhage, magnesium administration, NICU admission and 1-minute Apgar <7 were all significantly higher in the urgent-CD group. (3) The most common indications for urgent CD were contractions (42.6%) and PROM/PPROM (30.0%), (4) The median interval between planned and urgent CD was 5 days (0–51), and the median gestational age at which elective CD was scheduled was 38+2 weeks (37–40).

The optimal gestational age for a planned CD is a product of the maternal-neonatal balance between the possibility of preterm or early term morbidity and the possibility of urgent CD. Current recommendations [17] discourage non-indicated delivery before 39 gestational weeks because of its well established association with potential neonatal complications [14, 20]. However, some studies have shown that women who were scheduled for elective CD at 39 weeks were more likely than women scheduled for elective CD at 38 gestational weeks to require urgent CD [21, 22], placing them at increased risk of both maternal and fetal complications [23–27]. Overall, rates of transformation from planned to urgent CD in the literature range from 13% to 16% for CDs scheduled for 38 gestational weeks to 23% to 51% for CDs scheduled for 39 gestational weeks [21, 28, 29]. These values agree with the overall risk of 13% in our study, in which the median gestational age for planned CD was 38+3 weeks (37 to 40).

Chronic hypertension as well as hypertensive disorders of pregnancy were significantly more common among women in the urgent CD group. Hypertension by itself, in women without an indication for CD, has not been shown in previous studies to be a risk factor for CD. However, chronic hypertension is an established risk factor for preeclampsia [30, 31], one of many hypertensive disorders in pregnancy that are often managed by delivery. In these cases, delivery is by urgent CD when the disorder occurs at an early gestational age in patients with an unfavorable cervix or with a contraindication for vaginal delivery.

Women treated with antenatal corticosteroids were 3.2 times more likely to undergo urgent CD (OR 3.2, CI 2.4–4.5, Table 4). There are several possible explanations for this finding. First, studies have reported a significant decrease in the neonatal complication rate following antenatal corticosteroids treatment [32, 33], which makes it easier for the attending physician to decide on earlier delivery. Second, the very administration of antenatal corticosteroids indicates an underlying condition in which delivery, either spontaneous or iatrogenic, may be imminent. Hence, in women who have already received antenatal corticosteroids, the probability of there being an indication for urgent delivery is higher.

The indications for which the women were initially referred for elective CD varied. More women in the urgent-CD than the control group were scheduled for elective CD because of a contraindication for vaginal delivery. The odds ratio for undergoing urgent CD among women with a contraindication for vaginal delivery was 1.67 (95% CI 1.32–2.12, P<0.05) (Table 4). Some of the reasons for ruling out vaginal delivery, such as placenta previa, fetal growth restriction [34, 35], and malpresentation accompanied by PROM or /PPROM, are also independent risk factors for urgent CD.

Studies have shown that women with a history of multiple CDs are more likely to have adhesions and associated surgical complications [36–38]. Although 39 weeks has usually been reported as the optimal time for elective CD, there is some evidence that in women after two or more cesarean sections, the risk of maternal adverse outcome is higher for planned CD at 39 weeks compared to 38 weeks [21, 28]. Therefore, even in the absence of a clearcut indication for an earlier delivery there may be a tendency among physicians to schedule CD in these cases at an earlier gestational age to lower the chance of labor onset ahead of the schedule. If medically possible, in patients who present at odd hours, they are also likely to postpone these complex surgical procedures to the daytime when more experienced surgeons are available.

Neonates born via urgent CD had an increased risk of a 1-minute Apgar score <7 and of NICU admission. Mothers undergoing urgent CD had a higher risk for postpartum hemorrhage. These findings are supported by previous studies which showed poorer maternal and neonatal outcomes in urgent versus elective CDs [23–27, 39–41].

This study was limited by the retrospective design and the selection bias resulting from our policy to schedule CDs earlier in more surgically complex cases, lowering the chances that these women would enter spontaneous labor. The classification of indications for CD is debatable, as some indications include various groups in them. In addition, data on infant long-term outcome were lacking. The main strengths of the study were the inclusion of two population groups attending a single-center with uniform treatment protocols. Moreover, the large sample size was sufficient to detect significant differences in maternal and neonatal complications between the groups, such that we were able to investigate an issue that has not been previously addressed in the literature.

## Conclusion

To our knowledge, this is the first study to examine risk factors for urgent CD in women scheduled for elective CD. Significant risk factors for urgent CD were chronic hypertension, antenatal corticosteroids administration, and a contraindication for vaginal delivery as the reason for scheduling an elective CD. The timing of elective CD is based on a balance between maternal and neonatal factors and is set optimally in the majority of cases at 39 gestational weeks. However, in several subgroups of women who are prone to earlier delivery, an earlier gestational age might be considered.

## References

[1] World Health Organization. WHO recommendations non-clinical interventions to reduce unnecessary caesarean sections. WHO recommendations non-clinical interventions to reduce unnecessary caesarean sections. [WHO website] 2018. https://apps.who.int/iris/bitstream/handle/10665/275377/9789241550338-eng.pdf?ua=1. Accessed July 12, 2022.

[2] Betrán AP, Ye J, Moller AB, Zhang J, Gülmezoglu AM, Torloni MR. The increasing trend in caesarean section rates: global, regional and national estimates: 1990–2014. PLoS One 2016;11:e0148343. doi: 10.1371/journal.pone.0148343 26849801 PMC4743929

[3] Wells JC, Wibaek R, Poullas M. Global epidemiology of use of and disparities in caesarean sections. Lancet 2019;394:24–5. doi: 10.1016/S0140-6736(19)30715-9 31282355

[4] Boyle A, Reddy UM, Landy HJ, Huang CC, Driggers RW, Laughon SK. Primary cesarean delivery in the United States. Obstet Gynecol 2013;122:33–40. doi: 10.1097/AOG.0b013e3182952242 23743454 PMC3713634

[5] Barber EL, Lundsberg LS, Belanger K, Pettker CM, Funai EF, Illuzzi JL. Indications contributing to the increasing cesarean delivery rate. Obstet Gynecol 2011;118:29–38. doi: 10.1097/AOG.0b013e31821e5f65 21646928 PMC3751192

[6] Timofeev J, Reddy UM, Huang CC, Driggers RW, Landy HJ, Laughon SK. Obstetric complications, neonatal morbidity, and indications for cesarean delivery by maternal age. Obstet Gynecol 2013;122:1184–95. doi: 10.1097/AOG.0000000000000017 24201681 PMC4189107

[7] Resende MC, Santos L, Santos Silva I. Neonatal Morbidity in Term Newborns Born by Elective Cesarean Section. Acta Med Port 2015;28:601–7. 26667863

[8] Prediger B, Mathes T, Polus S, Glatt A, Bühn S, Schiermeier S, et al. A systematic review and time-response meta-analysis of the optimal timing of elective caesarean sections for best maternal and neonatal health outcomes. BMC Pregnancy Childbirth 2020;20:395. doi: 10.1186/s12884-020-03036-1 32641019 PMC7341650

[9] Sananès N, Haller L, Jochum F, Koch A, Lecointre L, Rozenberg P. Morbidity associated with planned cesarean deliveries performed before the scheduled date: A cohort study. Eur J Obstet Gynecol Reprod Biol. 2021 Sep;264:83–87. doi: 10.1016/j.ejogrb.2021.06.030 34284272

[10] Riskin A, Gonen R, Kugelman A, Maroun E, Ekhilevitch G. Does cesarean section before the scheduled date increase the risk of neonatal morbidity? Isr Med Assoc J. 2014 Sep;16(9):559–63. 25351013

[11] Wilmink FA, Hukkelhoven CW, Lunshof S, Mol BW, van der Post JA, Papatsonis DN. Neonatal outcome following elective cesarean section beyond 37 weeks of gestation: a 7-year retrospective analysis of a national registry. Am J Obstet Gynecol 2010;202:250.e1–8. doi: 10.1016/j.ajog.2010.01.052 20207243

[12] Nir V, Nadir E, Feldman M. Late better than early elective term Cesarean section. Acta Paediatr 2012;101:1054–7. doi: 10.1111/j.1651-2227.2012.02772.x 22758608

[13] Glavind J, Kindberg SF, Uldbjerg N, Khalil M, Møller AM, Mortensen BB, et al. Elective caesarean section at 38 weeks versus 39 weeks: neonatal and maternal outcomes in a randomised controlled trial. BJOG 2013;120:1123–32. doi: 10.1111/1471-0528.12278 23682628

[14] Tita AT, Landon MB, Spong CY, Lai Y, Leveno KJ, Varner MW, et al; Eunice Kennedy Shriver NICHD Maternal-Fetal Medicine Units Network. Timing of elective repeat cesarean delivery at term and neonatal outcomes. N Engl J Med 2009;360:111–20. doi: 10.1056/NEJMoa0803267 19129525 PMC2811696

[15] Armson BA. Is planned cesarean childbirth a safe alternative? CMAJ 2007;176:475–6. doi: 10.1503/cmaj.061724 17296960 PMC1800576

[16] Declercq E, Barger M, Cabral HJ, Evans SR, Kotelchuck M, Simon C, et al. Maternal outcomes associated with planned primary cesarean births compared with planned vaginal births. Obstet Gynecol 2007;109:669–77. doi: 10.1097/01.AOG.0000255668.20639.40 17329519

[17] American College of Obstetricians and Gynecologists’ Committee on Obstetric Practice, Society for Maternal-Fetal Medicine. Medically indicated late-preterm and early-term deliveries: ACOG Committee Opinion, Number 831. Obstet Gynecol 2021;138:e35–9. doi: 10.1097/AOG.0000000000004447 34259491

[18] NICE guideline. Caesarean birth [NICE website]. 2021. https://www.nice.org.uk/guidance/ng192/chapter/Recommendations#planned-caesarean-birth. Accessed July12, 2022.

[19] Dollberg S, Haklai Z, Mimouni FB, Gorfein I, Gordon ES. Birth weight standards in the live-born population in Israel. Isr Med Assoc J 2005;7:311–4. 15909464

[20] Clark SL, Miller DD, Belfort MA, Dildy GA, Frye DK, Meyers JA. Neonatal and maternal outcomes associated with elective term delivery. Am J Obstet Gynecol 2009;200:156.e1–4. doi: 10.1016/j.ajog.2008.08.068 19110225

[21] Melamed N, Hadar E, Keidar L, Peled Y, Wiznitzer A, Yogev Y. Timing of planned repeat cesarean delivery after two or more previous cesarean sections—risk for unplanned cesarean delivery and pregnancy outcome. J Matern Fetal Neonatal Med 2014;27:431–8. doi: 10.3109/14767058.2013.818130 23795868

[22] Glavind J, Henriksen TB, Kindberg SF, Uldbjerg N. Randomised trial of planned caesarean section prior to versus after 39 weeks: unscheduled deliveries and facility logistics—a secondary analysis. PLoS One 2013;8:e84744. doi: 10.1371/journal.pone.0084744 24376842 PMC3869904

[23] Pallasmaa N, Ekblad U, Aitokallio-Tallberg A, Uotila J, Raudaskoski T, Ulander V-M, et al. Cesarean delivery in Finland: maternal complications and obstetric risk factors. Acta Obstet Gynecol Scand 2010;89:896–902. doi: 10.3109/00016349.2010.487893 20583935

[24] Hawrylyshyn PA, Bernstein P, Papsin FR. Risk factors associated with infection following cesarean section. Am J Obstet Gynecol 1981;139:294–8. doi: 10.1016/0002-9378(81)90013-2 7468697

[25] Phipps MG, Watabe B, Clemons JL, Weitzen S, Myers DL. Risk factors for bladder injury during cesarean delivery. Obstet Gynecol 2005;105:156–60. doi: 10.1097/01.AOG.0000149150.93552.78 15625157

[26] Nielsen TF, Hökegård KH. Cesarean section and intraoperative surgical complications. Acta Obstet Gynecol Scand 1984;63:103–8. doi: 10.3109/00016348409154643 6730921

[27] Nielsen TF, Hökegård KH. Postoperative cesarean section morbidity: a prospective study. Am J Obstet Gynecol 1983;146:911–6. doi: 10.1016/0002-9378(83)90963-8 6881224

[28] Todumrong N, Somprasit C, Tanprasertkul C, Bhamarapravatana K, Suwannarurk K. A comparative study of the spontaneous labor rate in scheduled elective cesarean section at 38 weeks versus 39 weeks of gestation in parturient with previous cesarean section. J Med Assoc Thai 2016;99 Suppl 4:S37–41. 29916674

[29] Ramadan MK, Abdulrahim A, Itani SE, Hourani M, Mirza FG. Timing of an elective repeat cesarean delivery at term: addressing the controversy. J Clin Gynecol Obstet 2019;8:1–8.

[30] Sibai BM, Lindheimer M, Hauth J, Caritis S, VanDorsten P, Klebanoff M, et al. Risk factors for preeclampsia, abruptio placentae, and adverse neonatal outcomes among women with chronic hypertension. National Institute of Child Health and Human Development Network of Maternal-Fetal Medicine Units. N Engl J Med 1998;339:667–71. doi: 10.1056/NEJM199809033391004 9725924

[31] Rey E, Couturier A. The prognosis of pregnancy in women with chronic hypertension. Am J Obstet Gynecol 1994;171:410–6. doi: 10.1016/0002-9378(94)90276-3 8059820

[32] Roberts D, Brown J, Medley N, Dalziel SR. Antenatal corticosteroids for accelerating fetal lung maturation for women at risk of preterm birth. Cochrane Database Syst Rev 2017;3:CD004454. doi: 10.1002/14651858.CD004454.pub3 28321847 PMC6464568

[33] Gyamfi-Bannerman C, Thom EA, Blackwell SC, Tita ATN, Reddy UM, Saade GR, et al; NICHD Maternal–Fetal Medicine Units Network. Antenatal betamethasone for women at risk for late preterm delivery. N Engl J Med 2016;374:1311–20. doi: 10.1056/NEJMoa1516783 26842679 PMC4823164

[34] Baumfeld Y, Herskovitz R, Niv ZB, Mastrolia SA, Weintraub AY. Placenta associated pregnancy complications in pregnancies complicated with placenta previa. Taiwan J Obstet Gynecol 2017;56:331–5. doi: 10.1016/j.tjog.2017.04.012 28600043

[35] Sheiner E, Shoham-Vardi I, Hallak M, Hershkowitz R, Katz M, Mazor M. Placenta previa: obstetric risk factors and pregnancy outcome. J Matern Fetal Med 2001;10:414–9. doi: 10.1080/714052784 11798453

[36] Tulandi T, Agdi M, Zarei A, Miner L, Sikirica V. Adhesion development and morbidity after repeat cesarean delivery. Am J Obstet Gynecol 2009;201:56.e1–6. doi: 10.1016/j.ajog.2009.04.039 19576375

[37] Morales KJ, Gordon MC, Bates GW Jr. Postcesarean delivery adhesions associated with delayed delivery of infant. Am J Obstet Gynecol 2007;196:461.e1–6. doi: 10.1016/j.ajog.2006.12.017 17466702

[38] Lyell DJ. Adhesions and perioperative complications of repeat cesarean delivery. Am J Obstet Gynecol 2011;205(6 Suppl):S11–8. doi: 10.1016/j.ajog.2011.09.029 22114993

[39] Yang XJ, Sun SS. Comparison of maternal and fetal complications in elective and emergency cesarean section: a systematic review and meta-analysis. Arch Gynecol Obstet 2017;296:503–12. doi: 10.1007/s00404-017-4445-2 28681107

[40] Benzouina S, Boubkraoui Mel-M, Mrabet M, Chahid N, Kharbach A, El-Hassani A, et al. Fetal outcome in emergency versus elective cesarean sections at Souissi Maternity Hospital, Rabat, Morocco. Pan Afr Med J 2016;23:197. doi: 10.11604/pamj.2016.23.197.7401 27347286 PMC4907743

[41] Mölgg A, Jirecek S, Girtler V, Lehner R. Maternal and neonatal outcome for singleton and twin pregnancies in emergency cesarean section vs. urgent cesarean section in a retrospective evaluation from 2003–2012. Open J Obstet Gynecol 2014;04:881–8.

